# *Stenotrophomonas goyi *sp. nov., a novel bacterium associated with the alga *Chlamydomonas reinhardtii*

**DOI:** 10.12688/f1000research.134978.1

**Published:** 2023-10-18

**Authors:** María Jesus Torres, Neda Fakhimi, Alexandra Dubini, David González-Ballester

**Affiliations:** 1 Departamento de Bioquímica y Biología Molecular, Campus Universitario de Rabanales, Universidad de Cordoba, Córdoba, Andalusia, 14071, Spain; 2 Carnegie Institution for Science Department of Biosphere Sciences and Engineering, Stanford, California, 94305, USA

**Keywords:** algae, bacteria, consortia, cocultures, Chlamydomonas, Stenotrophomonas, vitamins, metabolic complementation

## Abstract

**Background**: A culture of the green algae *Chlamydomonas reinhardtii* was accidentally contaminated with three different bacteria in our laboratory facilities. This contaminated alga culture showed increased algal biohydrogen production. These three bacteria were independently isolated.

**Methods:** The chromosomic DNA of one of the isolated bacteria was extracted and sequenced using PacBio technology. Tentative genome annotation (RAST server) and phylogenetic trees analysis (TYGS server) were conducted. Diverse growth tests were assayed for the bacterium and for the alga-bacterium consortium.

**Results:** Phylogenetic analysis indicates that the bacterium is a novel member of the *Stenotrophomonas* genus that has been termed in this work as *S. goyi sp. nov.* A fully sequenced genome (4,487,389 base pairs) and its tentative annotation (4,147 genes) are provided. The genome information suggests that *S. goyi* sp. nov. is unable to use sulfate and nitrate as sulfur and nitrogen sources, respectively. Growth tests have confirmed the dependence on the sulfur-containing amino acids methionine and cysteine. *S.* *goyi sp. nov.* and *Chlamydomonas reinhardtii* can establish a mutualistic relationship when cocultured together.

**Conclusions**: *S. goyi* sp. nov. could be of interest for the design of biotechnological approaches based on the use of artificial microalgae-bacteria multispecies consortia that take advantage of the complementary metabolic capacities of their different microorganisms.

## Introduction

The first described species of the *Stenotrophomonas* genus was *S. maltophilia*, which was a Gram-negative bacterium originally named as *Pseudomonas maltophilia*, and later transferred in 1993 to the new genus *Stenotrophomonas*, which was solely composed of *S. maltophilia.* In 2001, this species was moved to the genus *Xanthomonas* before it was finally moved back again in 2017 to its own genus when *Stenotrophomonas pictorum* was identified ( Ryan *et al*., 2009; Wei *et al*., 2021). Currently, *Stenotrophomonas* is a genus comprising at least 19 validated species ( https://lpsn.dsmz.de/genus/stenotrophomonas) ( Parte *et al*., 2020). However, the molecular taxonomy of the genus is still somewhat unclear, and all its members are considered as “orphan species”. All *Stenotrophomonas* spp. have shown intraspecific heterogeneity with high phenotypic, metabolic, and ecological diversity ( Ryan *et al*., 2009).

The main reservoirs of *Stenotrophomonas* spp. are soil and plants, although they are ubiquitously present in different environments, including opportunistic human pathogens such as *S. maltophilia* ( Ryan *et al*., 2009).

*Stenotrophomonas* spp. show promising potential for different biotechnological applications. Some *Stenotrophomonas* spp. are of interest to agriculture due to their ability to promote growth in different plant species. Some *Stenotrophomonas* spp. are even capable of establishing symbiotic relationships with plants. This plant growth promotion is related to the capacity of some *Stenotrophomonas* spp. to produce the plant growth hormone indole-3-acetic acid (IAA), fix nitrogen, oxidate elemental sulfur (S) to sulfate, or biocontrol plant pathogens ( Banerjee and Yesmin, 2008; Park *et al*., 2005; Ryan *et al*., 2009; Suckstorff and Berg, 2003).

Moreover, they are also considered good candidates for bioremediation due to their tolerance to heavy metals and capability to metabolize a large variety of organic molecules, including phenolic and aromatic compounds ( Liu *et al*., 2007; Mora-Salguero *et al*., 2019; Pages *et al*., 2008; Ryan *et al*., 2009). Finally, some *Stenotrophomonas* spp. can synthetize useful bioproducts such as antimicrobial and enzymes of biotechnological interest ( Rivas-Garcia *et al*., 2022; Wolf *et al*., 2002).

Here we report the genome of *Stenotrophomonas goyi.* sp. nov. isolated from a contaminated microalgae ( *Chlamydomonas reinhardtii*) culture *.* This alga culture was simultaneously contaminated with *S. goyi, Microbacterium fakhimi* ( Fakhimi *et al*., 2023a) and *Bacillus cereus.* The metabolic interactions established between these four microorganisms are analyzed and discussed in a related publication where the ability of this multispecies consortium to sustain hydrogen production is highlighted ( Fakhimi *et al*., 2023b).

## Methods

### Isolation of Stenotrophomonas goyi sp. nov.

This study took place at Campus Universitario de Rabanales, Cordoba, Spain. *S. goyi* sp. nov. where it was isolated from a fortuitously contaminated *Chlamydomonas reinhardtii* culture in the laboratory. Initially, the *Chlamydomonas reinhardtii* culture was simultaneously contaminated with three different bacteria ( Fakhimi *et al*., 2023b). Individual members of this bacterial community were isolated by sequential rounds of plate streaking in Yeast Extract Mannitol (YEM) medium (handmade in our lab, described here), until three different types of bacterial colonies were visually identified. Colonies were grown separately, and the subsequent isolated DNA was used for PCR-amplification of their partial RNA 16S sequences. After sequencing, the three independently isolated bacteria were identified as members of the genus *Microbacterium*, *Stenotrophomonas*, and *Bacillus* ( Fakhimi *et al*., 2023b).

### Genome sequencing and assembling of S. goyi sp. nov.

DNA extraction and whole genome sequencing using PacBio (Pacific Biosciences) RS II Sequencing System (RRID:SCR_017988) were performed by SNPsaurus LLC ( https://www.snpsaurus.com/). Whole genome sequencing generated 102,238 reads yielding 832,209,774 bases for 166 read depth over the genome ( Table 1). Genome was assembled with Canu (RRID:SCR_015880) 1.7 ( Koren *et al*., 2017) generating a 4,487,389 pb circular genome. The genome completeness was checked by BUSCO 3.0.2 (RRID:SCR_015008) ( Manni *et al*., 2021) and was 94.6% complete, with 94.6% of the genome single copy and 0.0% duplicated. Any other prokaryotic contamination was discarded using ContEst16S 1.0 (RRID:SCR_000595) ( Lee *et al*., 2017).

**Table 1.** Main *de novo* sequencing and assembly statistics of *Stenotrophomonas goyi* sp. nov. genome.

Genome Size (pb)	Fold-coverage	GC content (%)	N° of contigs	Type	Plasmids
4,487,389	166	66.5	1	circular	No

### Phylogenetic analysis

Phylogenetic analyses were performed using the Type (Strain) Genome Server (TYGS) at Leibniz Institute DSMZ (German Collection of Microorganisms and Cell Cultures GmbH) ( Camacho *et al*., 2009; Farris, 1972; Kreft *et al*., 2017; Lagesen *et al*., 2007; Lefort *et al*., 2015; Meier-Kolthoff *et al*., 2022, 2013; Meier-Kolthoff and Göker, 2019; Ondov *et al*., 2016). Information on nomenclature, synonymy and associated taxonomic literature was provided by TYGS's sister database, the List of Prokaryotic names with Standing in Nomenclature (LPSN). Trees were inferred with FastME 2.1.6.1. Phylogenetic trees were drawn with iTOL (RRID:SCR_018174) ( Letunic and Bork, 2021).

### Annotation

Tentative annotation of the *S. goyi sp. nov.* genome was performed using the RAST tool kit, RASTtk (RRID:SCR_014606) at The Genome Annotation Service ( Brettin *et al*., 2015; Overbeek *et al*., 2014). BlastKOALA service was used to automatically assign K numbers to the predicted proteins, which allowed Kyoto Encyclopedia of Genes and Genomes (KEGG) orthology assignments, the putative characterization of individual gene functions, and the reconstruction of KEGG pathways ( Kanehisa *et al*., 2016). The PHAge Search Tool Enhanced Release (PHASTER) (RRID:SCR_005184) was used to locate potential phage sequences within the *S. goyi sp. nov.* genome ( Arndt *et al*., 2016)

### Bacterium growth media and culturing conditions

Bacterial precultures were grown on Yeast Extract Mannitol (YEM) or Luria Broth (LB) media (handmade in our lab). Some growth experiments were done in Mineral Medium (MM) ( Harris, 2008) supplemented with different nutrient sources (handmade in our lab). Tris-Acetate-Phosphate (TAP) medium ( Harris, 2008) (handmade in our lab, described here) was also used occasionally. In some experiments, a vitamin cocktail (riboflavin, 0.5 mg⋅L ^-1^; p-aminobenzoic acid, 0.1 mg⋅L ^-1^; nicotinic acid 0.1 mg⋅L ^-1^; pantothenic acid, 0.1 mg⋅L ^-1^; pyridoxine, 0.1 mg⋅L ^-1^; biotin, 0.001 mg⋅L ^-1^; vitamin B12, 0.001 mg⋅L ^-1^; thiamine, 0.001 mg⋅L ^-1^) was added to bacterial cultures. More specific details for each experiment can be found in the corresponding figure and table legends. All the bacterium cultures were incubated at 24-28°C and under continuous agitation (130 rpm).

### Coculturing algae and bacteria

*Chlamydomonas* cells were cultured for 3-4 days in TAP medium until mid-logarithmic growth phase, harvested by centrifugation (5.000 rpm for 5 min) and washed twice with fresh MM. Bacterial batch-cultures were incubated in TYM or LB medium until the Optical Density at 600 nm (OD _600_) reached 0.8-1, then harvested by centrifugation (12.000 rpm for 5 min) and washed twice with fresh MM. Algae and bacteria were cocultured in 250 mL flasks containing 100 mL of the corresponding medium. Alga-bacterium cocultures were set to initial chlorophyll concentration of 10 μg·mL ^−1^ for the alga and an initial OD _600_ of 0.1 for the bacterium. Algal and bacterial monocultures were used as controls. All cultures were incubated at 24°C with continuous agitation (120-140 rpm) and under continuous illumination (80 PPFD).

### Determinations of algal and bacterial growth

The algal growth was assessed in terms of chlorophyll content. Chlorophyll measurements were done by mixing 200 μL of the cultures with 800 μL of ethanol 100%. The mix was incubated at room temperature for 2-3 min, and afterward centrifuged for 1 min at 12.000 rpm. The supernatant was used to measure chlorophyll (a + b) spectrophotometrically (DU 800, Beckman Coulter) at 665 and 649 nm ( Wintermans and de Mots, 1965).

Bacterial growth in monocultures was estimated spectrophotometrically in terms of OD _600_ evolution (DU 800, Beckman Coulter). However, estimation of the bacterial growth in cocultures required bacterium cells separation from the alga cells. To do this, a customized Selective Centrifugal Sedimentation (SCS) approach was used. This approach consisted in finding the centrifugation parameters that led to maximize algal cell sedimentation while minimizing bacterial cell sedimentation ( Torres *et al*., 2022). Thus, measuring the OD of the supernatant after centrifugation can provide an estimation of the bacterial growth in the cocultures. To do this, the percentages of precipitated cells of each monoculture were calculated at different forces (from 100 to 500 x g) and times (1 and 2 min) using the measured OD before (A _BC_) and after (A _AC_) the centrifugation. Centrifugation at 200 x g for 1 min led to 87.9% of *Chlamydomonas* sedimentation, while only 2.1% of the bacterial cells dropped (meaning that 97.9% of the *S. goyi* cells remained in the supernatant). These parameters were chosen as a good compromise for SCS and used to evaluate the contribution of the bacteria to the OD in cocultures ( ^SCS^OD _600_).

## Results

### Identification of Stenotrophomonas goyi sp. nov.

**Figure 1.**
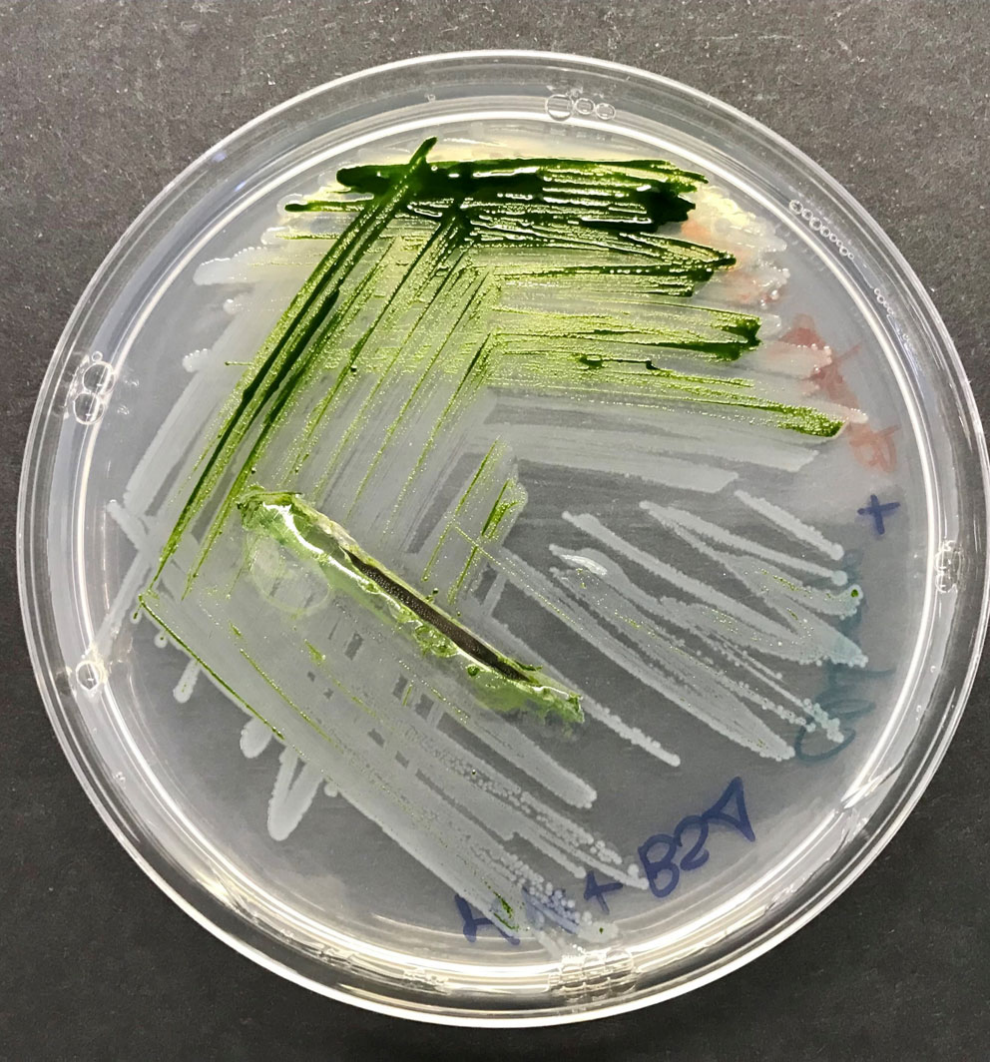
Plate with *Chlamydomonas reinhardtii* and *Stenotrophomonas goyi* sp. nov.

A fortuitous contaminated *Chlamydomonas reinhardtii* culture (strain 704; CC-3597; https://www.chlamycollection.org/) was studied due to its enhanced hydrogen production capability. This alga culture turned out to be contaminated with three different bacterial strains ( Fakhimi *et al*., 2023b), one of them consisting in a white-pigmented bacterium ( Figure 1). This bacterium was isolated after several rounds of plate streaking in TYM medium. First, partial PCR amplification and sequencing of the ribosomal 16S gene allowed the identification of this bacterium as a member of the *Stenotrophomonas* genus. Afterwards, the whole genome sequence was obtained. Genome assembling provided one single circular contig of 4,487,389 pb ( Table 1). No plasmids or extrachromosomal elements were identified.

The RAST server identified 4,147 genes (4,066 CDS + 81 rRNAs and tRNAs) ( Table 2). Out of these 4,066 CDS identified by RAST, 1,096 of them were in subsystems. Tentative genome annotation derived from the RAST server is available in **Supplemental Table 1** as *Extended data* ( González-Ballester *et al.*, 2023).

**Table 2.** Genome features of *Stenotrophomonas goyi* sp. nov. according to the RAST server. tRNA, transfer RNA; rRNA, ribosomal RNA.

Genes	CDS	tRNAs	rRNAs
4,147	4,066	71	10

**Figure 2.**
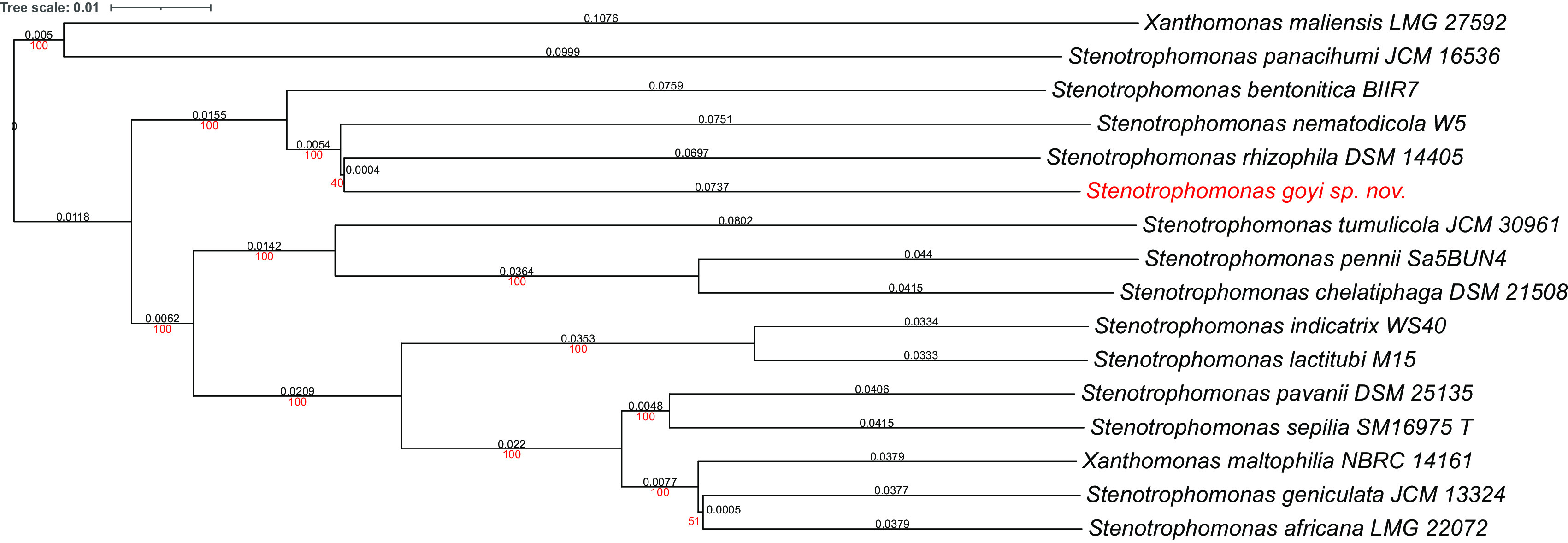
Phylogenetic tree for *Stenotrophomonas goyi* genome and related closest bacteria. Tree inferred with FastME 2.1.6.1 using the Genome BLAST Distance Phylogeny (GBDP) distances calculated from genome sequences. The branch lengths are scaled in terms of GBDP distance formula d5. The numbers above the branches are GBDP pseudo-bootstrap support values > 60% from 100 replications, with an average branch support of 91.6%. The tree was rooted at the midpoint. Branch lengths (black) and bootstraps (red) values are indicated. Genome sizes: 3,906,271–5,177,426 pb. Average δ statistics: 0.078 ( Holland *et al*., 2002). Phylogenetic tree drawn with iTOL ( Letunic and Bork, 2021).

**Figure 3.**
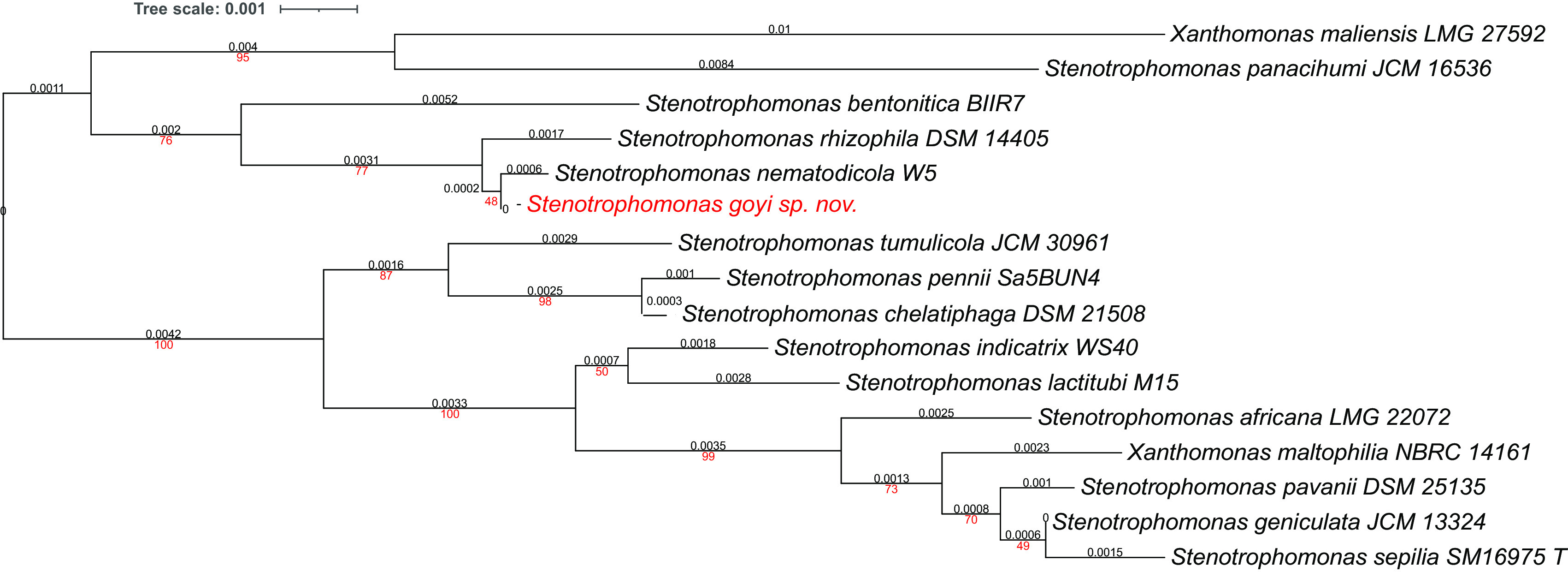
Phylogenetic tree for *Stenotrophomonas goyi* 16S rDNA and related closest bacteria. Tree inferred with FastME 2.1.6.1 using the Genome BLAST Distance Phylogeny (GBDP) distances calculated from 16S rDNA gene sequences. The branch lengths are scaled in terms of GBDP distance formula d5. The numbers above branches are GBDP pseudo-bootstrap support values > 60% from 100 replications, with an average branch support of 78.6%. The tree was rooted at the midpoint. Branch lengths (black) and bootstraps (red) values are indicated. RNA16S lengths: 1,385–1,535 pb. Average δ statistics: 0.236 ( Holland *et al*., 2002). Phylogenetic tree drawn with iTOL ( Letunic and Bork, 2021).

Phylogenetic analyses were performed with both, the whole genome ( Figure 2) and the inferred 16S rDNAs ( Figure 3). Pairwise comparisons with the closest type strains genomes are shown in Table 3. These phylogenetic analyses revealed that the sequenced genome belonged to a new *Stenotrophomonas* sp.; all dDDH values (d0, d4 and d6) were below 70% ( Meier-Kolthoff *et al*., 2013) ( Table 3). This new bacterial species was named as *Stenotrophomonas goyi* sp. nov. The closest related bacteria in terms of whole genome and 16S rDNA similarities were *Stenotrophomonas rhizophila* DSM 14405 and *Stenotrophomonas nematodicola* W5, respectively ( Figures 2 and 3). *S. goyi* sp. nov. genome was deposited in the NCBI as SUB12103906.

**Table 3.** Pairwise dDDH values between *S. goyi* sp. nov. and the closest type strains genomes. The digital DNA-DNA Hybridization (dDDH) values are provided along with their confidence intervals (C.I.) for the three different Genome BLAST Distance Phylogeny (GBDP) formulas: a) formula d0: length of all High-Scoring segment Pairs (HSPs) divided by total genome length; b) formula d4: sum of all identities found in HSPs divided by overall HSP length; formula d6: sum of all identities found in HSPs divided by total genome length ( Meier-Kolthoff *et al*., 2013).

Closest strains to Stenotrophomonas goyi sp. nov.	dDDH (d0, in %)	C.I. (d0, in %)	dDDH (d4, in %)	C.I. (d4, in %)	dDDH (d6, in %)	C.I. (d6, in %)	G+C content difference (in %)
*Stenotrophomonas rhizophila DSM 14405*	50,6	[47.2-54.0]	30,9	[28.6-33.5]	45	[42.0-48.1]	0,78
*Stenotrophomonas nematodicola W5*	48,9	[45.5-52.4]	29,9	[27.5-32.4]	43,4	[40.4-46.4]	0,82
*Stenotrophomonas bentonitica BIIR7*	40,9	[37.5-44.3]	28,9	[26.5-31.4]	37,1	[34.1-40.1]	0,05
*Xanthomonas maltophilia NBRC 14161*	36,2	[32.8-39.7]	24,8	[22.5-27.3]	32,3	[29.3-35.4]	0,34
*Stenotrophomonas sepilia SM16975 T*	37,4	[34.0-40.9]	24,6	[22.3-27.1]	33,1	[30.1-36.2]	0,06
*Stenotrophomonas lactitubi M15*	37,7	[34.3-41.1]	24,6	[22.3-27.1]	33,3	[30.3-36.4]	0,63
*Stenotrophomonas geniculata JCM 13324*	36,4	[33.0-39.9]	24,4	[22.1-26.9]	32,3	[29.4-35.4]	0,34
*Stenotrophomonas africana LMG 22072*	35	[31.6-38.5]	24,4	[22.0-26.8]	31,3	[28.4-34.4]	0,21
*Stenotrophomonas indicatrix WS40*	39,2	[35.8-42.7]	24,4	[22.0-26.8]	34,2	[31.3-37.3]	0,09
*Stenotrophomonas tumulicola JCM 30961*	33,1	[29.7-36.7]	24,4	[22.0-26.8]	29,9	[27.0-33.0]	0,9
*Stenotrophomonas pavanii DSM 25135*	38,6	[35.3-42.1]	24,3	[22.0-26.8]	33,8	[30.9-36.9]	0,87
*Stenotrophomonas chelatiphaga DSM 21508*	36,6	[33.3-40.1]	24,2	[21.9-26.6]	32,4	[29.5-35.5]	0,32
*Stenotrophomonas pennii Sa5BUN4*	35,9	[32.5-39.4]	24	[21.7-26.5]	31,8	[28.9-34.9]	0,08
*Stenotrophomonas panacihumi JCM 16536*	26,7	[23.4-30.4]	22,4	[20.1-24.8]	24,7	[21.9-27.8]	2,37
*Xanthomonas maliensis LMG 27592*	19,3	[16.1-22.9]	21,5	[19.2-23.9]	18,8	[16.1-21.8]	0,32

BlastKOALA ( Kanehisa *et al*., 2016) service allowed KEGG orthology assignments to characterize individual gene functions and reconstruct KEGG pathways of *S. goyi* genome ( **Supplemental Table 2;** *Extended data* ( González-Ballester *et al.*, 2023)). Some important pathways were either absent or incomplete in *S. goyi* sp. nov. including assimilation of nitrate (the whole assimilatory pathway is missing including nitrate transporters) and sulfate (only sulfite reductase is present). On the other hand, putative complete pathways for the glyoxylate cycle and biosynthesis of biotin, coenzyme A, pantothenate, riboflavin, tetrahydrofolate, glutathione, pyridoxal-P, lipoic acid, dTDP-L-rhamnose, UDP-N-acetyl-D-glucosamine, C5 isoprenoids, bacterial lipopolysaccharides, and antimicrobial proteins, among others, were present. Incomplete pathways for the degradation of aromatic compounds (including phenol, toluene, xylene, methylnaphthalene, 3-hydroxytoluene, and terephthalate) and myo-inositol biosynthesis, were also present.

Search with PHASTER ( Arndt *et al*., 2016) revealed one intact prophage (PHAGE_Erwini_phiEt88_NC_015295) located at position 753112-799783 of the *S. goyi* sp. nov. genome.

### Nutrient requirements of S. goyi sp. nov.

*S. goyi* sp. nov. showed no growth on MM, or in MM supplemented with different C sources (sucrose, glucose, lactose, mannitol, or glycerol) ( Table 4). The addition of vitamins to the MM supplemented with glucose or lactose did not support the bacterium growth either ( Table 4). However, the bacterium showed an excellent growth when cultivated in MM supplemented with yeast extract, tryptone, peptone or even Bovine Serum Albumin (BSA) ( Table 4), suggesting that this bacterium has a great capacity to use peptides/amino acids as C source, and probably also as N source. Moreover, the peptides/amino acids could also provide, in addition to C and N sources, other essential nutrients or even palliate potential amino acids auxotrophies. Note that MM medium has sulfate as only S source. As commented before, the genome of *S. goyi* sp. nov. is lacking a functional sulfate assimilation pathway. Thereby S-containing amino acids, such as cysteine and methionine, could support the growth in medium rich in peptides/amino acids.

**Table 4.** Growth of *S. goyi* sp. nov. on different nutrients. Mineral Medium (MM) was supplemented with different nutrients at 5 g·L ^-1^ each, but methanol and ethanol (5 ml·L ^-1^). For acetic acid, Tris-Acetate-Phosphate (TAP) medium was employed (1.05 g·L ^-1^ of acetic acid). Vitamins cocktail included riboflavin (0.5 mg⋅L ^-1^), p-aminobenzoic acid (0.1 mg⋅L ^-1^), nicotinic acid (0.1 mg⋅L ^-1^), pantothenic acid (0.1 mg⋅L ^-1^), pyridoxine (0.1 mg⋅L ^-1^), biotin (0.001 mg⋅L ^-1^), vitamin B12 (0.001 mg⋅L ^-1^), thiamine (0.001 mg⋅L ^-1^). ++, significant growth; +, poor growth; -, no growth.

Nutrients added	Growth
Sucrose	-
Lactose	-
Lactose + vitamins	-
Glucose	-
Glucose + vitamins	-
Mannitol	-
Lactic acid	-
Glycerol	-
Acetic acid (TAP medium)	-
Tryptone	++
Peptone	++
Yeast extract	++
BSA	++

**Figure 4.**
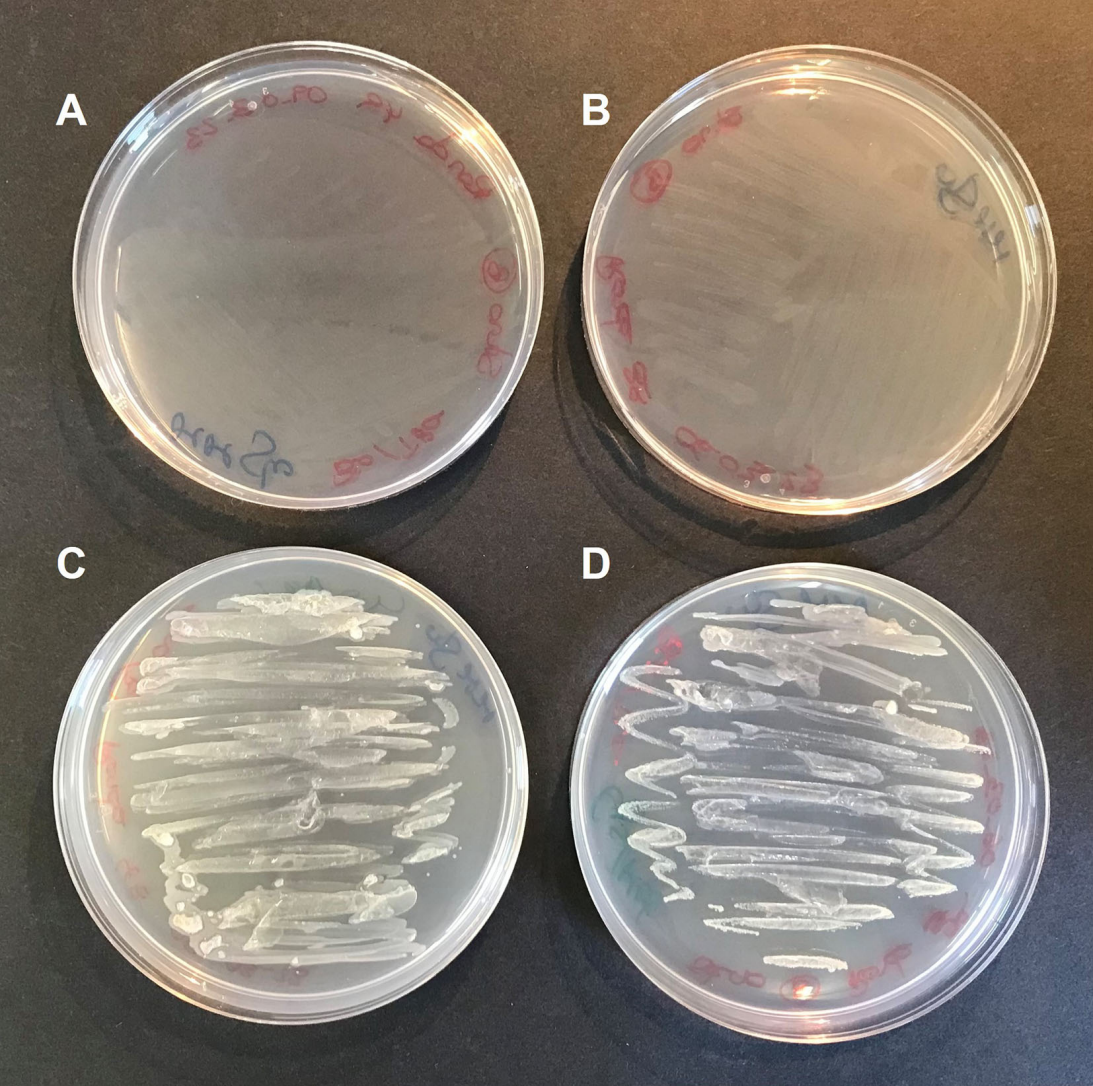
Cysteine and methionine requirements of *S. goyi* to grow. *S. goyi* sp. nov. was inoculated in: A) plates of Mineral Medium (MM) + glucose (5 g·L ^-1^); B) MM + glucose + biotin (0.001 mg·L ^-1^) + thiamine (0.001 mg·L ^-1^); C) MM + glucose + cysteine (4 mM) + methionine (4 mM); and D) MM + glucose + cysteine + methionine + biotin + thiamine.

To confirm this hypothesis, *S. goyi* sp. nov. was inoculated in plates of MM + glucose supplemented with different combinations of cysteine, methionine, biotin, and thiamine. Only plates containing cysteine and methionine supported the bacterial growth for several culturing rounds ( Figure 4). This result confirms the cysteine and methionine growth dependence of *S. goyi.* sp. nov. Cysteine and methionine could provide either a reduced S source or complement an auxotrophy for these two amino acids. Since *S. goyi* sp. nov. genome has complete pathways for all the essential amino acids, is more likely that cysteine and methionine are being used as reduced S sources. Similar results were found for *M. fakhimi*, where cysteine and methionine are required as S sources ( Fakhimi *et al*., 2023a).

*S. goyi* sp. nov. showed optimal growth between 24 and 32°C and pH 5-9 ( Table 5). Despite the presence of the complete multidrug resistance efflux pump MexJK-OprM in the genome ( Chuanchuen *et al*., 2005), no resistance to tetracycline, rifampicin, chloramphenicol and polymyxin (50 μg/mL each) was observed.

**Table 5.** Growth of *S. goyi* sp. nov. at different temperatures and pHs. Lysogeny broth (LB) medium was used in all the conditions.

Temperature °C	Growth	pH	Growth
10	-	3	-
15	+	4	+
20	++	5	+++
24	+++	6	+++
28	+++	7	+++
32	+++	8	+++
37	++	9	+++
42	-	10	+
		11	-

### Growth of S. goyi sp. nov -C. reinhardtii consortium

Torres *et al.*, (2022) reported that cocultures of *S. goyi* sp. nov. (published as *Stenotrophomonas* sp.) and *C. reinhardtii* promoted the growth of the microalga (nearly doubled) when incubated in MM supplemented with glucose and mannitol, but not when supplemented with acetic acid ( Torres *et al*., 2022).

**Figure 5.**
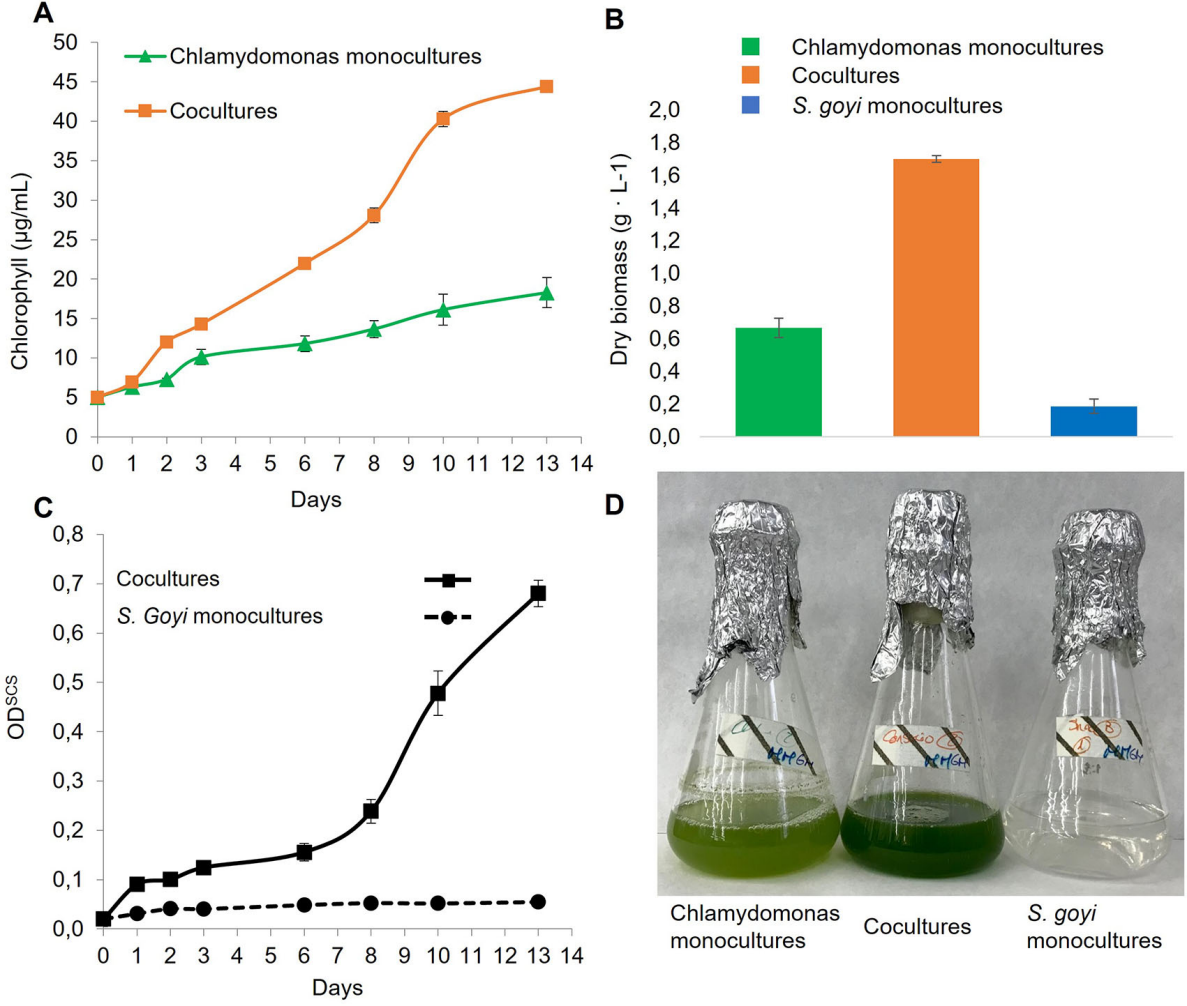
*S. goyi* sp. nov. and *C. reinhardtii* growth in consortium. *S. goyi-C. reinhardtii* consortium, and respective control monocultures, were incubated in Mineral Medium (MM) supplemented with glucose (5 g·L ^-1^) and mannitol (5 g·L ^-1^). A) Chlorophyll content; B) dry weight biomass after 13 days; C) bacterial growth in terms of OD ^SCS^; D) actual picture of the cultures after 13 days.

Here, it was also checked if the bacterium also benefited when co-cultivated with C *. reinhardtii* in glucose- and mannitol-containing media. First, we observed that the chlorophyll content in the cocultures was 2.4 times higher than in the *C. reinhardtii* monocultures after 13 days ( Figure 5A), which is in accordance with previous results ( Torres *et al*., 2022). Additionally, the dry biomass resulting from the consortia was 2.2 times higher than the sum of the respective monocultures ( Figure 5B). Finally, the growth of the bacterium in cocultures was very efficient, unlike *S. goyi* sp. nov. monocultures ( Figure 5C).

These results indicate that *S. goyi* sp. nov. and *C. reinhardtii* can establish a mutualistic relationship when incubated in sugars-containing media. On the one hand, *S. goyi* sp. nov. can greatly support the growth of the *C. reinhardtii* in media supplemented with glucose or mannitol, which are two carbon sources that the alga cannot utilize. Likely, this growth promotion is due to the release of acetate and/or CO _2_ from the bacteria after the sugar fermentation. Acetate is the sole organic carbon source that *C. reinhardtii* can utilize during heterotrophic/mixotrophic growth ( Chaiboonchoe *et al*., 2014). On the other hand, *S. goyi*, sp. nov. can grow in media without amino acids/peptides supplementation when cocultured with *C. reinhardtii*, suggesting that the alga must provide some essential nutrients for the bacterium. Reduced S forms excreted by the alga ( *e.g.*, cysteine or methionine) could potentially explain the bacterium growth in the consortium.

## Discussion and conclusions

*Stenotrophomonas* spp. are common constituents of the rhizosphere, and their potential for agricultural biotechnology is arising. However, their association with algae is poorly explored. Most plant growth-promoting bacteria (PGPB) are firstly identified in the rhizosphere and in association with plants. However, many PGPB are then also often commonly found in association with algae. This is likely reflecting that the kind of relationships established between bacteria and plants are similar to the relationships between bacteria and algae. This could potentially be the case for *Stenotrophomonas* spp., although the relative poor taxonomic curation and heterogeneity of the genus may prevent the tracking of its association with algae.

Some *Stenotrophomonas* spp. show a limited nutritional range, while others are capable of metabolic versatility ( Ryan *et al*., 2009). *S. goyi* sp. nov. is unable to grow in the absence of a source of peptides/amino acids, which imply that in natural ecosystems it may rely on other microorganisms to obtain essential nutrients. As stated before, *S. goyi sp. nov.* is unable to use sulfate as S source. The peptides/amino acids are likely providing S-reduced forms (such as cysteine and methionine) to *S. goyi sp. nov.*

*Stenotrophomonas goyi* sp. nov. was isolated from an alga culture ( *C. reinhardtii*) that showed an enhanced capacity to produce hydrogen and biomass when incubated in mannitol and yeast extract containing medium ( Fakhimi *et al*., 2023b). This algal culture was simultaneously contaminated with two other bacteria: *Microbacterium fakhimi* and *Bacillus cereus.* Out of the three bacteria, *M. fakhimi* was the main responsible for the enhanced algal hydrogen production. However, *C. reinhardtii-M. fakhimi* cocultures were unable to produce hydrogen and biomass concomitantly. In addition to *M. fakhimi*, the presence of *S. goyi sp. nov. and B. cereus* in the cocultures was needed to produce joinlty hydrogen and algal biomass ( Fakhimi *et al*., 2023b), which stresses the biotechnological interest of *S. goyi sp. nov.*

*M. fakhimi* showed auxotrophy for biotin and thiamine, and like *S. goyi sp. nov.* was unable to grow on inorganic S sources ( Fakhimi *et al*., 2023a). In this multispecies association, *S. goyi sp. nov.* and *C. reinhardtii* could alleviate the auxotrophy of *M. fakhimi* sp. nov. for biotin and thiamine. *S. goyi sp.* *nov.* could also provide ammonium derived from the mineralization of the amino acids to the alga. On the other hand, the alga could provide S-reduced sources such as cysteine and methionine for *S. goyi* sp. nov. and *M. fakhimi.* In any case, this multispecies association was mutually beneficial and prevented an excessive bacterial growth in cocultures, which could be one of the main drawbacks when algae-bacteria cocultures are used for biotechnological applications.

Nevertheless, the precise metabolic relationships established in this multispecies consortium that led to the extension of the *C. reinhardtii* cells viability during hydrogen production condition is not yet unraveled and need to be further investigated.

## Ethical considerations

Not applicable.

## Data Availability

Spanish Type Culture Collection (CECT): *Stenotrophomonas goyi sp. nov.* bacterium. Accession number CECT30764; https://www.cect.org/vstrn.php?lan=en&cect=30764 ( Universidad de Cordoba, 2022). NCBI Gene: *Stenotrophomonas goyi sp. nov.* genome sequence. Accession number CP116871; https://identifiers.org/ncbi/insdc:CP124620 ( Fakhimi *et al.*, 2023). Zenodo: Supplemental Files, https://doi.org/10.5281/zenodo.8091305 ( González-Ballester *et al.*, 2023). This project contains the following extended data:
- Supplemental Table 1 [Tentative annotation of the *S. goyi sp. nov.* genome] - Supplemental Table 2 [KEGG orthology assignments] Data are available under the terms of the Creative Commons Attribution 4.0 International license (CC-BY 4.0).
