# Supplementary material for: Stenotrophomonas goyi sp. nov., a novel bacterium associated with the alga Chlamydomonas reinhardtii
Source: F1000Res. 2023 Dec 14;12:1373. Originally published 2023 Oct 18. [Version 3] doi: 10.12688/f1000research.134978.3 (PMC10682605; doi:10.12688/f1000research.134978.3)
Supplement: Peer review history

# Reviewer report: Reviewer response for version 2

**DOI:** 10.5256/f1000research.157820.r224973

**Published:** 2023-11-27

**Authors:** Gergely Maroti

**Affiliations:** 1 Institute of Plant Biology, Biological Research Centre, Szeged, Hungary

The manuscript describes the characteristics of a novel *Stenotrophomonas* sp. bacterium isolated from a complex algal-bacterial association. The genome description is clear and concise, the nutrient requirements of the bacterium were also clearly determined.

 The mutualistic growth promoting effects were proven experimentally. The potential molecular mechanisms behind the observed effects are not shown or hypothesized, however, it is not the goal of this manuscript.

 The conclusions are correct, the application of multispecies consortia including bacteria and green algae is a promising way for biological hydrogen production in an economically viably manner.

Is the work clearly and accurately presented and does it cite the current literature?

Yes

If applicable, is the statistical analysis and its interpretation appropriate?

Yes

Are all the source data underlying the results available to ensure full reproducibility?

Yes

Is the study design appropriate and is the work technically sound?

Yes

Are the conclusions drawn adequately supported by the results?

Yes

Are sufficient details of methods and analysis provided to allow replication by others?

Yes

Reviewer Expertise:

Algal biotechnology

I confirm that I have read this submission and believe that I have an appropriate level of expertise to confirm that it is of an acceptable scientific standard.

# Reviewer report: Reviewer response for version 2

**DOI:** 10.5256/f1000research.157820.r224972

**Published:** 2023-11-27

**Authors:** Rosa Leon-Bañares

**Affiliations:** 1 Laboratory of Biochemistry, Faculty of Experimental Sciences, Marine International Campus of Excellence and REMSMA, University of Huelva, Huelva, Andalusia, Spain

The paper is clearly presented and describes an interesting study, which is detailed presented. In this paper, the authors describe the characterization of a new bacterial species of the genus Stenotrophomonas, found as contaminant in a culture of the chlorophyte Chlamydomonas reinhardtii. The bacterial genome has been sequenced and tentatively annotated. Phylogenetic trees based on both whole genome and 16S rDNA sequences were stablished. The  growth of the new bacteria, either with or without the microalga, was evaluated.

 Functional annotation of the genome of the new bacteria reveled that some important pathways are not present in S. goyi genome. This data, beside several growth test carried out with aminoacids and /or peptide hydrolysates make the authors suggest that peptides and amino acids are a good C source, and probably also a good N or S source, for S. goyi, which can not grow in minimal medium not supplemented with aminoacids or peptides. Addition of aminoacid/peptides or co-cultivation with the microalga is necessary for the growth of this bacterial. Moreover, the microalga growth rate is practically doubled when cultured in the presence of the bacteria. The authors propose the establishment of a mutualistic relationship between C. reinhardtii and the bacteria, in which the bacteria would provide a carbon source (acetate or CO2 obtained from bacterial sugar fermentation)  to the microalgae and the bacteria would obtain S-aminoacids excreted by the alga.

 The authors suggest that many microalgal-associated bacteria can promote higher plant growth, being the characterization of this algal-bacterial consortium of interest to identify potent plant growth-promoting bacteria.

 Suggestions:

- The assessment of the bacterial growth by determination of optical density is adequate for pure cultures, but it is difficult to carry out in mixed algal-bacterial cultures. The authors have optimized and validated a selective centrifugal sedimentation approach to separate bacteria and microalgae. Although the approach seems to work well, I would suggest determination of colony forming units (CFU) to follow bacterial growth in future occasions. In may experience, the sedimentation properties of the microalga can change along the growth cycle 
- Indicate in the legend of Figure 1, that shows a plate with Chlamydomonas reinhardtii and Stenotrophomonas goyi sp. nov., the culture medium used. Is it bacterial or algal growth médium? Is it MM or has been supplemented with sugars? 

Is the work clearly and accurately presented and does it cite the current literature?

Yes

If applicable, is the statistical analysis and its interpretation appropriate?

Not applicable

Are all the source data underlying the results available to ensure full reproducibility?

Yes

Is the study design appropriate and is the work technically sound?

Yes

Are the conclusions drawn adequately supported by the results?

Yes

Are sufficient details of methods and analysis provided to allow replication by others?

Yes

Reviewer Expertise:

Biochemistry and Biotechnology of microalgae

I confirm that I have read this submission and believe that I have an appropriate level of expertise to confirm that it is of an acceptable scientific standard.
